# Bioactive Foods Decrease Liver and Brain Alterations Induced by a High-Fat-Sucrose Diet through Restoration of Gut Microbiota and Antioxidant Enzymes

**DOI:** 10.3390/nu14010022

**Published:** 2021-12-22

**Authors:** Tauqeerunnisa Syeda, Mónica Sánchez-Tapia, Itzel Orta, Omar Granados-Portillo, Lizbeth Pérez-Jimenez, Juan-de-Dios Rodríguez-Callejas, Samuel Toribio, Maria-del-Carmen Silva-Lucero, Ana-Leonor Rivera, Armando R. Tovar, Nimbe Torres, Claudia Perez-Cruz

**Affiliations:** 1 Laboratorio de Neuroplasticidad y Neurodegeneración, Departamento de Farmacología, CINVESTAV, Mexico City 07360, Mexico; tauqeer.unnisa1@gmail.com (T.S.); lizagos1@hotmail.com (L.P.-J.); juan_316hov@hotmail.com (J.-d.-D.R.-C.); herecomes_sam@hotmail.com (S.T.); carmenaguila10@hotmail.com (M.-d.-C.S.-L.); 2 Departamento de Fisiología de la Nutrición, Instituto Nacional de Ciencias Médicas y Nutrición Salvador Zubirán, Mexico City 14080, Mexico; qfbmoniktc@gmail.com (M.S.-T.); itzzel1300@gmail.com (I.O.); ograpo@yahoo.com (O.G.-P.); armando.tovarp@incmnsz.mx (A.R.T.); 3 Instituto de Ciencias Nucleares y Centro de Ciencias de la Complejidad, Universidad Nacional Autónoma de México, Mexico City 04510, Mexico; ana.rivera@nucleares.unam.mx

**Keywords:** bioactive foods, gut microbiota, hepatic antioxidant enzymes, cognitive function

## Abstract

Obesity is associated with cognitive deficit and liver alterations; however, it remains unclear whether a combination of functional foods could reverse cognitive damage and to what extent it would be associated with changes in gut microbiota and liver. With this aim, male Wistar rats were fed a high-fat-5%sucrose diet (HFS) for 4 mo. And were then fed for 1 mo. with bioactive foods. At the end of this period, liver, serum, feces, intestine, and brain samples were taken. Body composition, energy expenditure, LPS, hormones, intraperitoneal glucose tolerance test, behavioral tests, and gut microbiota were evaluated. We showed that male rats fed high-fat-sucrose diet developed gut microbiota dysbiosis, increased in body fat, decreased antioxidant activity, decreased brain neuropeptide Y, increased the number of astrocytes and activated microglia, along with reduced spine density associated with deficits in working memory. Ingestion of a combination of nopal, soy protein, curcumin, and chia seed oil (bioactive foods) for three months was associated with an increase in a cluster of bacteria with anti-inflammatory capacity, a decrease in serum LPS levels and an increase in serum eicosapentaenoic acid (EPA) with neuroprotective properties. In the liver, ingestion of bioactive food significantly increased antioxidant enzymes, decreased lipogenesis, reduced inflammation mediated by the TLR4-TNFα pathway along with a decrease in body fat, glucose intolerance, and metabolic inflexibility. Finally, neuroinflammation in the brain was reduced and working memory improved. Our study demonstrates that consumption of bioactive foods was associated with reduced liver, brain, and gut microbiota alterations in obese rats.

## 1. Introduction

Studies in individuals and animals have demonstrated that consumption of a high fat and sugar diet causes inflammation which spread into the brain, affecting key components of cognition [1]. Several factors have been involved in the development of cognitive dysfunction, including dysbiosis in the gut microbiota [2], increase in oxidative stress in the central nervous system [3], and possibly other metabolic abnormalities in other organs of the body, particularly in the liver. In obesity, which is often associated with consumption of a high fat and sugar diet, an imbalance in the gut microbiota was found to be associated with impaired gut barrier function and translocation of lipopolysaccharide (LPS) and inflammation in various tissues, such as liver, adipose, and brain tissue [4,5,6]. Furthermore, a crosstalk between adipose tissue and the brain was suggested to contribute to cognitive deficits [6]. Several dietary guidelines have focused on the consumption of antioxidant-rich plant foods, which may be effective in maintaining cognitive health [3]. Thus, the purpose of the present work was to develop a combination of functional foods that included nopal, soy protein, turmeric, and chia seed oil (bioactive foods) to modulate gut microbiota and enhance antioxidant activity to improve cognitive function. In this study, we investigated whether consumption of an HFS diet produces dysbiosis of the gut microbiota, alters body composition and proinflammatory markers, decreases hepatic antioxidant enzymes, and impairs glucose and energy metabolism, brain structure, and cognitive function. Furthermore, we examined whether ingestion of bioactive foods (BF) can reverse these disturbances.

## 2. Materials and Methods

### 2.1. Study Design

Male Wistar rats aged 5–7 weeks were obtained from the National Institute of Medical Sciences and Nutrition. The animals were housed in individual cages, with access to food and water *ad libitum*, (12 h/12 h light–dark cycle, 20 °C, and 40–50% relative humidity). The protocol of this study was approved by the Bioethics Committee of the Instituto Nacional de Ciencias Médicas y Nutrición Salvador Zubirán, Mexico City (CINVA1444). The study consisted in two phases: (1) Obesity-induction: during the first 4 months, rats were randomized into two groups; (i) control rats (C, *n* = 8) fed a control diet prepared according to the American Institute of Nutrition recommendations (AIN-93) [4], and (ii) a high fat-sucrose diet (HFS) consisting of a high fat diet and 5% sucrose added to the drinking water (HFS, *n* = 32). At the fourth month, memory performance was assessed by T-Maze and novel object recognition test (NOR), followed by metabolic parameter measurements in plasma including glucose, triglycerides, and cholesterol levels. (2) Intervention phase: HFS group was divided into 4 groups: HFS diet (HFS, *n* = 8), HFS + bioactive food (HFS + BF, *n* = 8), HFS switched to control diet (HFS → C, *n* = 8), and HFS switched to control diet plus BF (HFS → C + BF, *n*= 8), over the course of three months. The C group continued consuming the AIN-93 diet (C, *n* = 8). BF replaced some components of the AIN-93 diet with dried nopal, soy protein, chia seed oil, and turmeric. However, both diets offer similar kcal/g (Appendix A, Table A1. Diet composition). Once a week, bottles were replaced with fresh solutions. Animal weight, and food consumption were recorded every other day during the protocol. At the end of this period, liver, serum, feces, intestine, and brain samples were immediately collected and stored at −70 °C.

### 2.2. Serum Biochemical Parameters

A total of one day after the last behavioral tests, animals were killed by cervical decapitation previous anesthesia. Blood was extracted by cardiac puncture and serum was obtained and stored a −70 °C until analysis. Serum parameters including glucose, triglyceride (TG), and total cholesterol were measured using an enzymatic-photometric assay with a Cobas c111 analyzer (Roche Applied Science). Insulin (ELISA kit Alpco Diagnostics, Salem, MA, USA), LPS (Cloud-Clone Corp, Houston, TX, USA), leptin (rat leptin ELISA kit, St Charles, MO, USA) and GIP (Merck-Millipore rat/mouse GIP (total) ELISA, Bellerice, MA, USA) were measured by ELISA.

### 2.3. Energy Expenditure

Rats were placed in a non-invasive in vivo calorimetric chamber of an Oxymax open circuit indirect calorimeter (Oxymax, Columbus Instruments, Columbus, OH, USA) to assess energy expenditure using measurements of oxygen consumption and carbon dioxide production for 24 h as previously described [5]. The respiratory exchange ratio (RER), which represents the ratio between the volume of CO_2_ produced by the body and the amount of O_2_ consumed, was assessed. A low RER (0.70) reflects predominant fat oxidation, whereas a high RER (1.00) is indicative of glucose oxidation.

### 2.4. Intraperitoneal Glucose Tolerance Test

Intraperitoneal glucose tolerance test (ipGTT) was determined as previously reported [6] by the administration of an intraperitoneal injection of a glucose load (2 g/kg body weight) in fasted rats. The area under the curve (AUC) was calculated using the trapezoid rule.

### 2.5. Behavioral Evaluation

A total of three days before the end of the first and second stages of the experimental design, novel object recognition (NOR) test and the T-maze test were performed according to previous descriptions [7,8]. For NOR, the discrimination index (DI) was calculated as: (time exploring the novel object − time exploring the familiar object) / (time exploring novel object + familiar object) × 100. For the T-maze, three trials were given on each day (from 09.00–11.00 hs), for two days, with a total of 6 trials per animal.

### 2.6. Body Composition

The body composition of each animal was evaluated at the end of the study using magnetic resonance imaging (EchoMRI, Echo Medical Systems, Houston, TX, USA). The scanning was performed by presenting the animals in a thin-walled plastic cylinder with a cylindrical plastic insert that limited animal movement. In the cylinder, the animals were briefly subjected to a low-intensity electromagnetic field (0.05 Tesla) for 2 min.

### 2.7. Gene Expression Analysis

The total RNA was extracted from the frozen livers of rats using TRIzol reagent (Invitrogen, Carlsbad, CA, USA), and cDNA synthesis was performed with M-MLV reverse transcriptase enzyme and oligo(dT) 12–18 primer (Invitrogen, Carlsbad, CA, USA). The mRNA levels were measured by real-time quantitative PCR using a LightCycler^®^ 480 SYBR Green I Master (Roche Applied Science, Mannheim, Germany) or LightCycler^®^ 480 master probes. The PCR assays for each analyzed gene were performed in triplicate, with 12 ng of reverse-transcriptase product each in 96-well optical plates using a LightCycler^®^ 480 Instrument (Roche Applied Science, Mannheim, Germany) in accordance with the manufacturer’s instructions. Ribosomal m36B4 protein (m36B4) or β_2_ microglobulin (B2M) mRNA were used as invariant controls. The relative expression was calculated as previously reported [9].

### 2.8. Microbiota Analysis

Fresh fecal samples collected at the end of the study were immediately frozen and stored at −70 °C until use. Bacterial DNA content was extracted using the QIAamp DNA Mini Kit (Qiagen, Valencia, CA, USA) according to the manufacturer’s instructions. MiSeq platform was used for the sequencing of the samples of the regions V3 and V4 of the 16S rRNA gene as previously described [6]. Total species diversity was determined by alpha diversity measurements (Shannon).

### 2.9. Brain Tissue Preparation

Shortly after rats were killed by cervical decapitation, brains were immediately collected. Brain was divided into two halves. Medial prefrontal cortex (PFC) (from Bregma AP: 3.70–2.20) was divided into left- and right-hemispheres. Left-PFC was snap frozen in liquid nitrogen and stored at −70 °C until processing. Right-PFC was post-fixed in 4% PFA for 4 h (4 °C) for dendritic spine analysis (diolistic labelling). The right brain region containing the arcuate nucleus (Bregma AP: −2.12 to −3.00) was post-fixed with 4% PFA for 72 h for immunofluorescence analysis.

### 2.10. Western-Blot Analysis

Tissues were homogenized in RIPA buffer (150 mM sodium chloride, 1.0% NP-40 or Triton X-100, 0.5% sodium deoxycholate, 0.1% sodium dodecyl sulphate, 50 mM Tris, pH 8.0) (10% weight/volume). Tissue samples were separated on SDS-PAGE (10–12%), and transferred onto a polyvinylidene difluoride membrane. Blots were incubated with the following primary antibodies: BDNF (1:250), Abcam, Cat. No. ab203573, RRID: AB_2631315), TLR4 (1:1500), Santa Cruz, sc-293072, RRID: AB_10611320), TLR2 (1:1000), Santa Cruz, sc-21760, NRF2 (1:5000), Santa Cruz, sc-722, TNFα (1:10,000), Abcam, Cat No ab205587, occludin (1:50,000), Abcam, Cat. No ab167161, p-NF-κB (1:10,000), Abcam, Cat No ab86299. Membranes were washed with TBS-T (TBS + 0.05% Tween20), and then incubated with a secondary antibody linked to horseradish peroxidase. As a loading control, α-actin (1:1000) or GAPDH (1:50,000) was used. Immunoreactive bands were visualized using Chemidoc (Bio rad XRS + SYSTEM). The western blots were performed at least 3 times using independent blots. Densitometry analysis was performed using NIH Image J software. Values were normalized with actin or GAPDH and expressed as fold increase.

### 2.11. Diolistic Labelling

Right-PFC was used to evaluate dendritic spine density according to previous descriptions [10,11]. Briefly, 80 µm brain slices were immediately shot with tungsten particles (1.2 µm) (Bio-Rad, Hercules, CA, USA) covered with DiL (1,1′-Dioctadecyl-3,3,3′,3′ Tetramethylindo-carbocyanine Perchlorate, Invitrogen, Cat. Num. D282 DiI) by use of a Helius Gene Gun (Bio-Rad, Hercules, CA, USA). Dendritic spines were classified into three different types according to NeuroStudio classification (NIH, Free Software), and as previously reported [10,11].

### 2.12. Immunohistochemistry

Brains were immersed in 30% sucrose in PBS for 4 days for cryoprotection. Coronal slices (40 μm thickness) were processed for immunofluorescence according to previous descriptions [8,10,11,12]. Slices were incubated with the following primary antibodies: anti-NYP (1:2000; Rabbit/IgG), anti-Glial Fibrillary Acidic Protein (GFAP, 1:500; Mouse/IgGMillipore, Cat. No. 3670, RRID: AB_561049), anti-iba1 (Iba1, 1:500; Rabbit/IgG, Wako Chemicals, Cat. No. 019-19741, RRID: AB_839504), all diluted in PBS with 5% horse serum for 48 h at 4 °C. Tissue was washed with 0.2% PBS-triton, and incubated with respective secondary antibodies in 0.2% PBS-triton. Control sections were processed without the primary antibody.

### 2.13. Image Acquisition and Morphometry

Bright-field images were obtained for NPY-immunolabeling with a Nikon Eclipse 80i light microscope equipped with a 10× objective. Fluorescent labelling (GFAP and iba1 staining) was assessed under a laser scanning microscope (Leica TCS-SP8) with optimized pinhole diameter under a 40× objective. NYP-immunoreactivity (ir) was analyzed in one image of the dorsal dentate gyrus and one image of arcuate nucleus (Bregma AP −2.30 to −3.60) per brain section, in at least 3 sections from 3 different animals, per group. The number of GFAP- and iba1- positive cells were quantified in at least 4 images of the PFC in at least 6 animals per group. The total number of astrocytes and microglia per region was calculated as (number of cells/number of images) × single image area (0.15 mm^2^). Microglia phenotype classification was based on previous reports [12,13]. The number of activated or inactivated microglia multiplied by 100 and divided by the total number of microglia per region cells yield the percentage of microglia.

### 2.14. Statistical Analysis

Statistical analysis was performed by one-way ANOVA followed by Tukey’s post-hoc test, using Prism 6.0 software (GraphPad, San Diego, CA, USA). Results are expressed and plotted as mean ± S.D. Differences were considered significant when *p* < 0.05. Microbial sequence data were pooled for OTUs comparison and taxonomic abundance analysis. Community diversity was determined by the number of OTUs and beta diversity, measured by UniFrac unweighted and weighted distance matrices in QIIME software v1.9.0. Analysis of similarities was performed using ANOSIM and permutational multivariate analysis of variance was performed using ADONIS. Differences in the relative abundance at the species levels was performed by using Linear Discriminant analysis (LDA) to determine the OTUs most likely to explain differences between treatment groups with the tool LEfSe in the Galaxy platform [14].

### 2.15. Bioinformatic Analysis

For taxonomic composition analysis, Custom C# and python scripts in the Quantitative Insights Into Microbial Ecology (QIIME) software pipeline 1.9 were used to process the sequencing files [15]. The sequence outputs were filtered for low-quality sequences. Sequences were checked for chimaeras with Gold.fa, and chimeric sequences were filtered out. The analysis started by clustering sequences within a percent sequence similarity into operational taxonomic units (OTUs). OTUs picking was performed with the tool set from QIIME, using the Usearch method [16]. OTUs were picked against the GreenGenes v.13.9 database. A total of ninety-seven percent of the OTUS were selected from the database. Species richness (Observed, Chao1) and alpha diversity measurements (Shannon) were calculated. Weighted and unweighted UniFrac distances were used to perform the principal coordinate analysis (PCoA). The diversity was measured by UniFrac unweighted and weighted distances matrices in QIIME. ANOSIM, a permutational multivariate analysis of variance, was used to determine statistically significant clustering of groups based upon microbiota structure distances. 

### 2.16. Principal Component Analysis (PCA)

We carried out a PCA to extract the important information from a multivariate matrix constructed from 55 variables. For the analysis PCA, the Origin Pro version 2021b package was applied over the normalizing data (taking out the media and scaling by the standard deviation of the data) to find the correlation matrix, diagonalizing it to produce the eigenvalues that were sorted in descending order, and we compared the eigenvectors to obtain the changes in importance of the different variables.

## 3. Results

### 3.1. Body Weight, Body Composition, and Energy Expenditure

**Figure 1:**
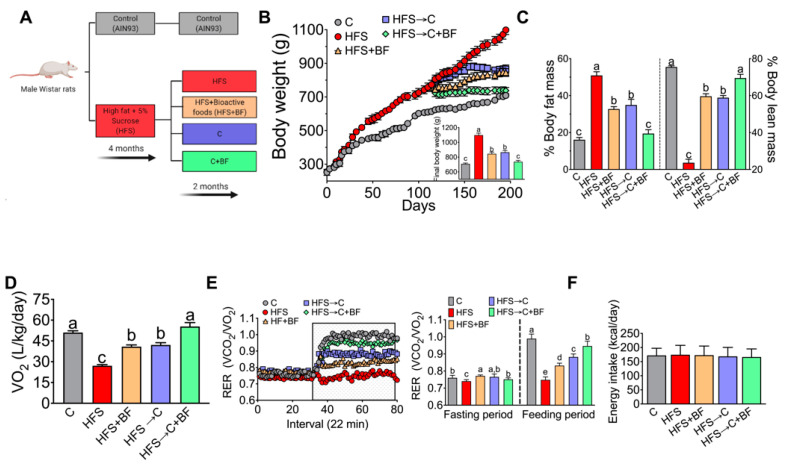
Bioactive foods decreased body weight and increased energy expenditure. (**A**) Experimental design, (**B**) Body weight gain, (**C**) Body composition, (**D**) Energy expenditure, (**E**) Respiratory exchange ratio (RER) and (**F**) Energy intake in rats fed control (**C**) or high fat diet + 5% sucrose (HFS) with or without bioactive foods (BF). Data are expressed as the mean ± SD. Mean values with different letters show statistical differences between each other (a > b > c > d). Data were analyzed by one-way ANOVA followed by Tukey post-hoc test. Results were considered statistically significant at *p* < 0.05. Panel A was created with BioRender.com (accessed date 19 December 2021).

To determine the effects of bioactive foods on body weight and body composition, we measured these variables after the diet-induced obesity stage (4 months) and at the end of the second stage after the different dietary intervention for three months (Figure 1A). After obesity induction phase, the rats fed HFS diet showed a significant increase in body weight gain by 24.2% with respect to the rats fed C diet. Interestingly, this weight gain was reversed with addition of bioactive foods by 23% with respect to HFS group; this reduction was faster than the group HFS → C diet (21.2%), however the combination of C diet with bioactive foods resulted in a 32.8% of decreased in body weight (Figure 1B). In order to evaluate if the changes in body weight were related to body composition, we measured body fat and body lean mass. The results revealed that the group fed HFS diet had a 2-fold increase in body fat relative to the C group. However, the rats fed HFS + BF diet showed a significant decrease in body fat by 35.7% with respect to the HFS group, and this pattern was similar in the HFS → C group with a decrease of 31.4%; however, the group fed the HFS → C + BF diet reached similar values to the control group with a 61.8% loss of body fat with respect to the HFS group (Figure 1C). With respect to lean body mass, the HFS group lost 68.7% of lean mass compared to C group, while the HFS + BF group only lost 21%; results were similar to those of the HFS → C group (22%), while the group fed C + BF lost only 7.9% of lean mass compared to the C group (Figure 1C). These results were related to the changes observed in energy expenditure, as the group HFS → C + BF and C group had very similar VO_2_ values (51–54 (L/kg/day), whereas the HFS group had a significant decrease in VO_2_ (27 L/kg/day) compared to the C group. Interestingly, energy expenditure was partially improved in the HFS + BF and HFS → C groups with an increase of 51% and 55% compared to rats fed HFS (Figure 1D). Thus, the dietary strategy of control diet and BF foods almost reversed the metabolic inflexibility (Figure 1E). It is important to mention that these changes in weight, body composition and energy expenditure were not influenced by caloric intake, which was similar in all groups (Figure 1F).

### 3.2. Glucose Tolerance and Insulin Sensitivity

**Figure 2:**
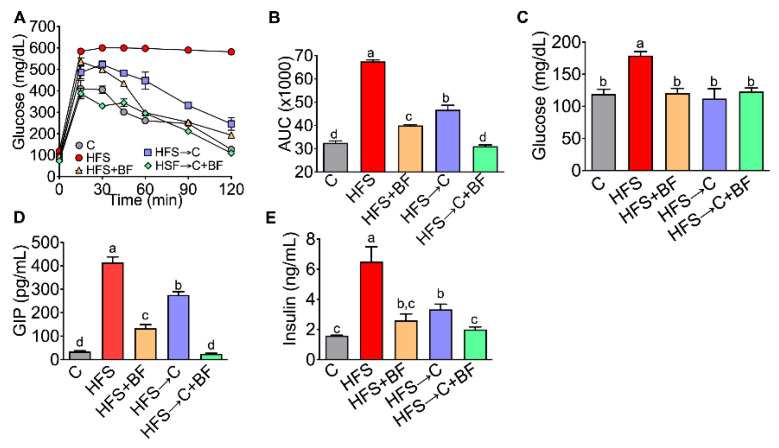
Consumption of bioactive foods improves glucose intolerance. (**A**) Glucose tolerance test, (**B**) Area under curve, (**C**) Serum glucose, (**D**) Serum Gastric inhibitor polypeptide (GIP), and (**E**) Serum insulin in rats fed control (**C**) or high fat diet + 5% sucrose (HFS) with or without bioactive food. Data are expressed as the mean ± SD. Mean values with different letters show statistical differences between each other (a > b > c > d). Data were analyzed by one-way ANOVA followed by Tukey post-hoc test. Results were considered statistically significant at *p* < 0.05.

Changes in body composition and energy expenditure are associated with abnormalities in glucose metabolism; for this reason, glucose tolerance was evaluated. The results revealed that the HFS group showed a significant glucose intolerance, because postprandial blood glucose values after 2 h did not return to baseline values, generating an area under the curve that increased by 107% with respect to the C group. The HFS → C group improved glucose tolerance by decreasing AUC by 30.7%. Interestingly, the addition of bioactive foods even in presence of HFS produced a significant decrease in AUC by 40.7%, and the combination of C diet and bioactive foods reduced the AUC by 54%, similar to the C group (Figure 2A,B). The fasting glucose was comparable in all groups except for the HFS group, which showed an increase of 50.2% compared to C group (Figure 2C). Because we observed important changes in glucose tolerance, we then studied the circulating levels of hormones such as GIP and insulin involved in inflammation and insulin resistance [17]. Our data showed a significant increase in GIP in the HFS group by 11- fold compared to the C group; the HFS → C and HFS + BF groups had a significant reduction of GIP values by 33.3% and 67.8%, respectively. Interestingly, rats fed HFS → C + BF showed a similar GIP concentration to that of the C group (Figure 2D). Insulin concentration had a similar pattern than GIP, where rats fed HFS showed a 3-fold increase with respect C group, whereas the HFS → C group had a reduced insulin concentration by 48.8% compared to HFS group. Notably, the groups with bioactive foods did not have significant differences to the C group (Figure 2E).

### 3.3. Gut Microbiota and Microbial α-Diversity

**Figure 3:**
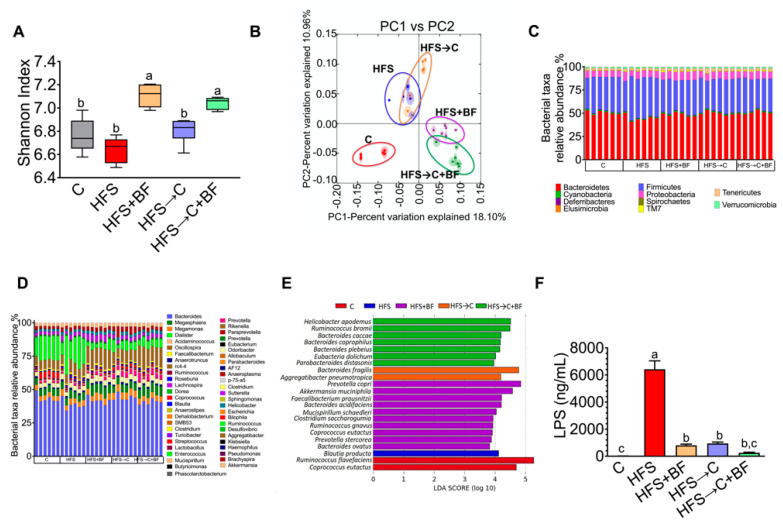
Consumption of bioactive foods decreased metabolic endotoxemia and increased gut microbiota diversity. (**A**) Alpha diversity by Shannon index, (**B**) Principal component analysis of gut microbiota, Relative abundance at (**C**) Phylum level and (**D**) Genus level, (**E**) Lineal discriminatory analysis at species level and (**F**) Serum lipopolysaccharide concentrations in rats fed control (C), high fat diet + 5% sucrose (HFS) with or without bioactive foods (BF). Data are expressed as the mean ± SD. Mean values with different letters show statistical differences between each other. Results were considered statistically significant at *p* < 0.05.

It has been widely reported that high fat diets alter gut microbiota composition. To examine whether bioactive foods could mitigate HFS-induced gut microbiota dysbiosis, we analyzed the taxonomic bacterial composition in fecal samples. The alpha diversity estimated by Shannon index showed a significant increase in α-diversity in the groups fed bioactive foods with respect to the C or the HFS groups (Figure 3A). Clustering the bacterial communities using principal components analysis (PCoA) revealed a clear dissimilarity between the groups fed bioactive foods and those fed C or HFS diets, indicating that the consumption of the combination of different bioactive foods modified the gut microbiota (Figure 3B). At the phylum level, there was a significant decrease in the Bacteroidetes in the HFS group and a significant increase in the bacterial phylum in the HFS → C + BF. The Proteobacteria group increased in the HFS → C and HFS + BF with respect to the C groups (Figure 3C). At the genus level, the presence of the bioactive foods significantly increased *Coprococcus, Ruminococcus,* and *Clostridium* (Figure 3D). At species level, rats fed HFS showed a pronounced abundance of *Blautia producta* compared to the rest of the groups. The addition of bioactive foods in the HFS diet significantly increased *Prevotella copri*, *Akkermansia muciniphila*, *Faecalibacterium prausnitzii*, *Bacteroides acidifaciens*, *Mucispirillum schaedleri*, *Clostridium sacharogumia*, *Ruminococcus gnavus*, *Coprococcus eutactus*, *Prevotella stercorea* and *Bacteroides ovatus.* However, the addition of the C diet significantly reduced all these bacteria (Figure 3E). Interestingly, the low-grade inflammation mediated by LPS was significantly reduced by 82.5%, 87.5% and 96.1% in the groups fed HFS → C, HFS + BF and HFS → C + BF, respectively, indicating that the addition of bioactive foods significantly reduces the metabolic endotoxemia by modification of a specific group of bacteria in the gut (Figure 3F).

### 3.4. TNFα Production and Occludin Abundance in the Colon

**Figure 4:**
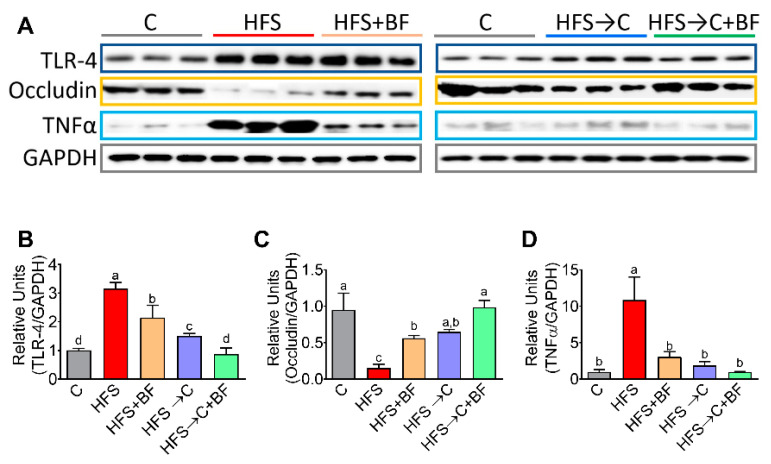
Gut barrier is improved with bioactive foods consumption. (**A**) Western blot analysis and (**B**–**D**) densitometric analysis of intestinal TLR-4, occludin and TNFα of rats fed control (**C**) or high fat diet + 5% sucrose (HFS) with or without bioactive foods. Data are expressed as the mean ± SD. Mean values with different letters show statistical differences between each other (a > b > c > d). Data were analyzed by one-way ANOVA followed by Tukey post-hoc test. Results were considered statistically significant at *p* < 0.05.

To assess whether the bioactive foods were able to modify the inflammatory process due to the stimulation of toll-like receptor 4 (TLR4) by LPS, we measured the abundance of TLR4, the proinflammatory cytokine TNF α and occludin involved in the formation of tight junctions in the colon. We found that the consumption of bioactive foods decreased to some extent the abundance of TLR4 and TNFα, and increased the abundance of occludin in the colon compared with the HFS group; however, the addition of C diet or C + BF completely reversed the inflammatory effect produced by the HFS diet (Figure 4A–D).

### 3.5. TNFα Release Mediated by TLR4 and pNF−κB

**Figure 5:**
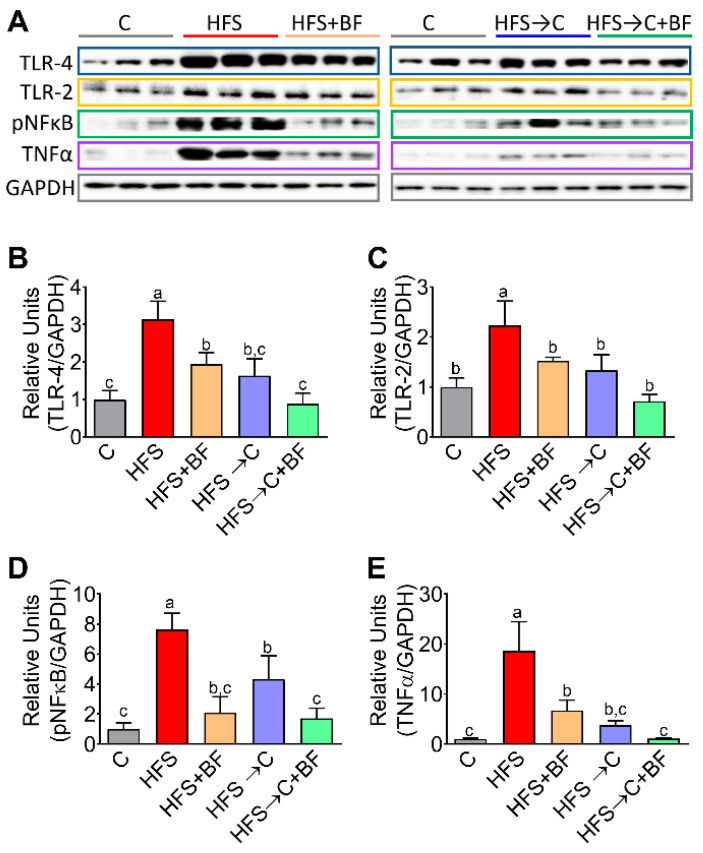
Liver inflammation decreased by consumption of bioactive foods. (**A**) Western blot analysis, (**B**) Protein abundance by (**B**) TLR-4, (**C**) TLR-2, (**D**) pNFκB and (**E**) TNFα in liver of rats fed control (**C**) or high fat diet+5% sucrose (HFS) with or without bioactive. Data are expressed as the mean ± SD. Mean values with different letters show statistical differences between each other. Data was analyzed by one-way ANOVA fol-lowed by Tukey post-hoc test. Results were considered statistically significant at *p* < 0.05.

Consumption of an HFS diet results in a loss of intestinal barrier integrity with a consequential increase in bacterial translocation of LPS released by Gram (-) bacteria into the liver via the gut–liver axis. Circulating LPS could initiate an intracellular signaling cascade through TLR4 or TLR2 in the liver. We measured TLR4 and TLR2 abundance in the liver, as well as the phosphorylation of the transcription factor NF-κB involved in the release of proinflammatory cytokines. We observed that addition of bioactive foods to the HFS diet significantly decreased the abundance of TLR4, TLR2, pNF-κB and TNFα with respect to the HFS diet at the same extent than HFS → C group. The combination of C diet and bioactive foods were more effective in decreasing inflammatory markers; however, bioactive foods were more effective in reducing NF-κB phosphorylation than the C diet alone (Figure 5A–E).

### 3.6. Expression of Lipogenic Genes

**Figure 6:**
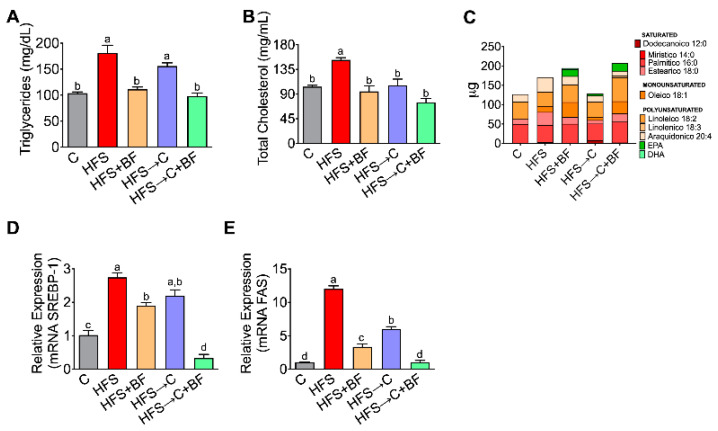
Consumption of bioactive foods decreased lipogenesis and improved lipid serum profile. (**A**) Serum triglycerides, (**B**) Serum total cholesterol, (**C**) Fatty acids serum profile, relative hepatic expression by (**D**) SREBP-1, and (**E**) FAS in rats fed control (**C**) or high fat diet + 5% sucrose (HFS) with or without bioactive foods. Data are expressed as the mean ± SD. Mean values with different letters show statistical differences between each other. Data were analyzed by one-way ANOVA followed by Tukey post-hoc test. Results were considered statistically significant at *p* < 0.05.

It has been demonstrated that TLR4 regulates obesity-induced inflammation and lipogenesis [18]; for this reason, we explored the lipid profile after the consumption of HFS diet or bioactive foods. Consumption of HFS diet significantly increased serum triglycerides and cholesterol concentration, and this was accompanied by an increase in the gene expression of the hepatic lipogenic genes sterol regulatory element binding-1 (SREBP-1) and fatty acid synthase (FAS). Interestingly, bioactive foods, even when an HFS diet was consumed, were more effective than C diet in lowering serum triglycerides, total cholesterol, hepatic SREBP-1, and FAS. On the other hand, the combination of bioactive foods and C diet further decreased SREBP-1 and FAS gene expression (Figure 6A–E). Growing evidence suggests the importance of omega-3 fatty acid intake in brain function; for this reason, we measured the serum fatty acids profile after the consumption of bioactive foods, due to the high concentration of linolenic acid in chia oil, which is an essential precursor of EPA. We observed that the consumption of a bioactive foods significantly increased eicosapentanoic acid (EPA; 20:5), which is considered as a neuroprotective metabolite (Figure 6C).

### 3.7. Hepatic Antioxidant System

**Figure 7:**
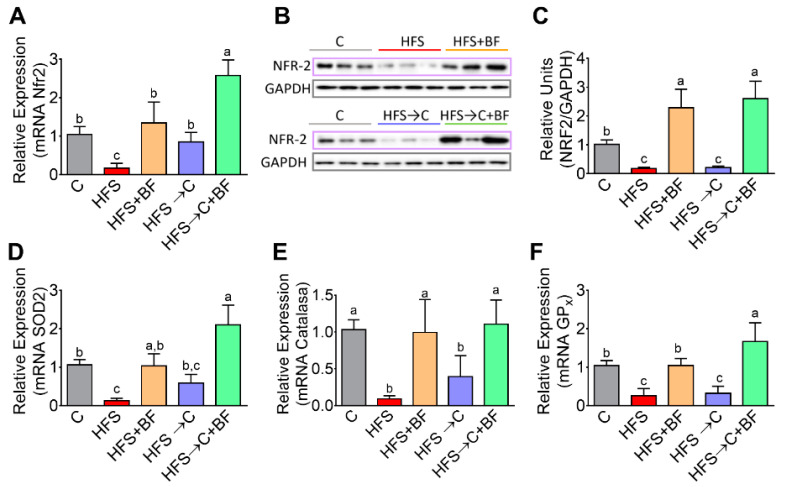
Hepatic antioxidant enzymes increased by consumption of bioactive foods. (**A**) Relative expression of mRNA abundance of Nfr2, (**B**) protein abundance of Nfr2 assessed by western blot analysis, and (**C**) densitometric analysis, relative expression of mRNA abundance of (**D**) Superoxide dismutase 2, (**E**) Catalase, and (**F**) Glutathione peroxidase in liver of rats fed control (**C**) or high fat diet + 5% sucrose (HFS) with or without bioactive foods. Data are expressed as the mean ± SD. Mean values with different letters show statistical differences between each other. Data were analyzed by one-way ANOVA followed by Tukey post-hoc test. Results were considered statistically significant at *p* < 0.05.

Growing evidence suggests that the gut–liver–brain axis is involved in the development of many diseases. Interestingly, oxidative stress is also a key player in the pathogenesis of neurological disorders; for this reason, we evaluated whether the combination of bioactive foods could modulate the antioxidant system in the liver. We found that consumption of bioactive foods in the HFS diet or C + BF diet significantly increased the transcription factor nuclear factor erythroid (NFR-2) involved in the regulation of gene expression of antioxidant enzymes. We observed that the consumption of bioactive foods even in the presence of an HFS diet, but not in the C group, significantly increased by 125% the protein abundance of NFR-2 in liver, with respect to the C group. The same pattern was observed for the antioxidant enzymes SOD2, catalase, and GPx, (Figure 7A–F).

### 3.8. Working Memory and Object Recognition

**Figure 8:**
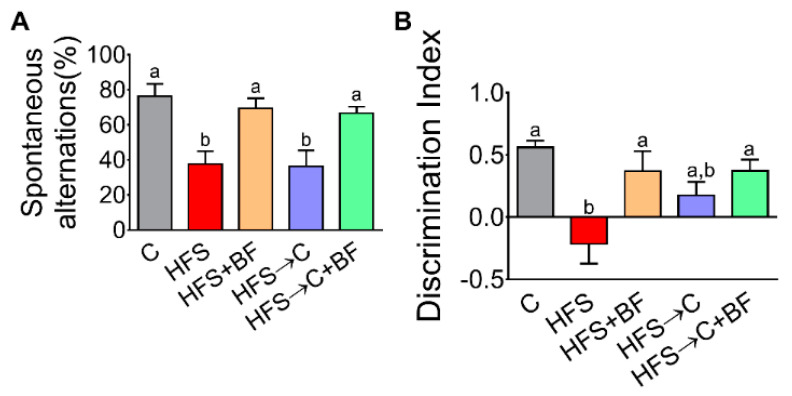
Consumption of bioactive foods improved spatial and working memory. (**A**) Spontaneous alternation by T-maze test and (**B**) discrimination index by novel object recognition (NOR) test in rats fed control (**C**) or high fat diet + 5% sucrose (HFS) with or without bioactive foods. Data are expressed as the mean ± SD. Mean values with different letters show statistical differences between each other. Data were analyzed by one-way ANOVA followed by Tukey post-hoc test. Results were considered statistically significant at *p* < 0.05.

Taking our current data into account, we evaluated whether bioactive foods containing antioxidants could mitigate the cognitive deficits observed in HFS rats. Working-memory and object recognition memory were evaluated by the T-maze and NOR tests, respectively. The percentage of spontaneous alterations in the T-maze was lower in HFS and HFS → C rats compared to C, indicating memory deficits caused by long-term HFS intake (4 months). These memory impairments were reversed by the addition of bioactive foods to the diets (Figure 8A). A lower discrimination index (DI) in the NOR test was observed in the HFS group compared to C, while the dietary intervention with bioactive foods, even in the presence of HFS diet, significantly increased DI values (Figure 8B).

### 3.9. Number of NYP-Positive Cells in Dentate Gyrus and BDFN Levels in the PFC

**Figure 9:**
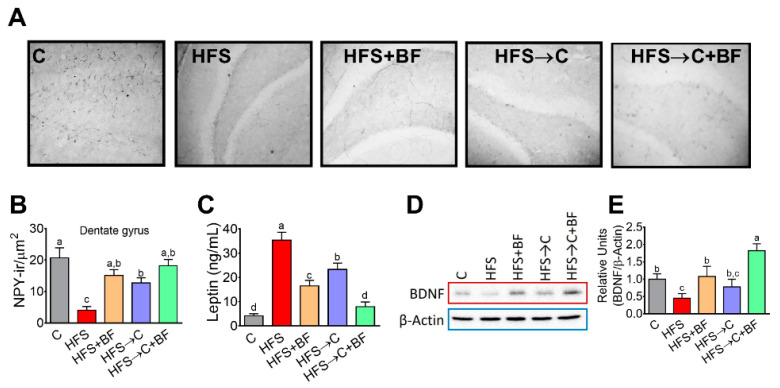
Neuropeptide Y in the dentate gyrus of the hippocampus were increased with bioactive foods. (**A**) NPY-ir in dentate gyrus, (**B**) quantification of NPY-ir, (**C**) serum leptin concentration (**D**) western blot analysis, and (**E**) protein abundance of BNDF in prefrontal cortex of rats fed control (**C**) or high fat diet + 5% sucrose (HFS) with or without bioactive foods. Data are expressed as the mean ± SD. Mean values with different letters show statistical differences between each other. Data were analyzed by one-way ANOVA followed by Tukey post-hoc test. Results were considered statistically significant at *p* < 0.05.

To assess whether HFS diet cause alteration in NYP cells located in cognitive-associated areas, we analyzed NYP-immunoreactivity (ir) in the dentate gyrus of the hippocampus. HFS rats showed a reduced NYP-ir compared to C group, but it was recovered after all dietary interventions (Figure 9A,B). Rats fed HFS showed a significant increase in leptin concentration (Figure 9C) associated with low NYP-ir production. Interestingly, the group fed bioactive foods containing chia seed oil composed mainly of omega 3 fatty acids significantly reduced leptin concentration, indicating that the type of fat reversed the hyperleptinemia (Figure 9C). Brain-derived neurotrophic factor (BDNF) contributes to memory consolidation and energy homeostasis. In the present work, we observed that the group fed HFS rats had a significantly reduced abundance of BDNF in the PFC, and bioactive foods ingestion increased those values even in the presence of HFS, probably due to the omega 3 fatty acid contained in the chia oil (Figure 9D,E).

### 3.10. Number of Dendritic Spines in the PFC

**Figure 10:**
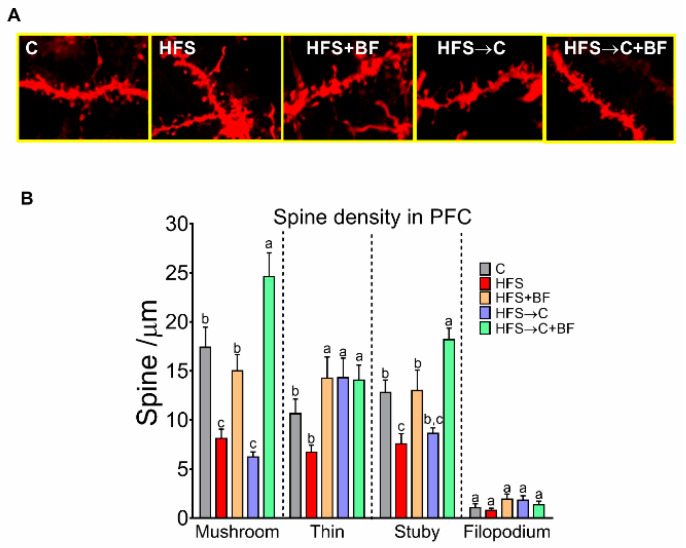
Dendric spine density increased in the prefrontal cortex with bioactive foods consumption. (**A**) Representative images of dendritic segments from neurons analyzed by NeuronStudio Software (NIH), (**B**) mushroom, thin, stubby, and filopodia spine density in prefrontal cortex of rats fed control (**C**) or high fat diet + 5% sucrose (HFS) with or without bioactive foods. Data are expressed as the mean ± SD. Mean values with different letters show statistical differences between each other. Data were analyzed by one-way ANOVA followed by Tukey post-hoc test. Results were considered statistically significant at *p* < 0.05.

As bioactive food induced significant memory improvements accompanied by increases in BDFN levels in the PFC, we aimed to quantify the number of mushroom, thin, and stubby spines in the right-PFC. There was a decreased density of mushroom spines in the HFS and HF-C group compared to C group. HFS → C + BF group showed the highest amount of mushroom spines compared to all groups. The density of thin spines increased in HFS + BF, HFS → C and HFS → C + BF compared to C and HF rats. The density of stubby spines was lower in HFS rats compared to C, but higher in HFS → C + BF compared to HFS and HFS → C + BF. No significant differences were observed in the number of filopodia (Figure 10A,B).

### 3.11. Neuroinflammation in the PFC

**Figure 11:**
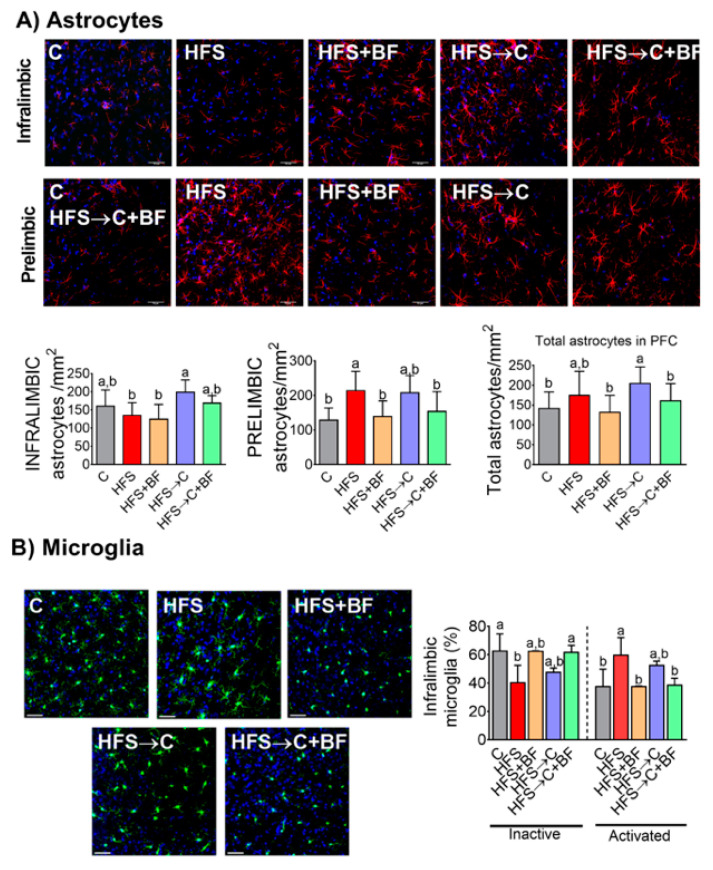
Astrocytes and microglia in prefrontal cortex were modified by bioactive foods. (**A**) Representative images of astrocytes in infralimbic and the prelimbic areas of prefrontal cortex and quantification and (**B**) representative images of microglia in infralimbic area of prefrontal cortex and quantification of activated or inactivated cells of rats fed control (**C**) or high fat diet + 5% sucrose (HFS) with or without bioactive foods. Data are expressed as the mean ± SD. Mean values with different letters show statistical differences between each other. Data were analyzed by one-way ANOVA followed by Tukey post-hoc test. Results were considered statistically significant at *p* < 0.05.

Due to the increased metabolic endotoxemia (LPS) and systemic inflammation markers in HFS rats, we aimed to determine whether bioactive food might impede glial cells activation in the PFC. The number of GFAP-positive astrocytes in the PFC was increased in HFS and HFS→C rats compared to C, but it remained stable in HFS → BF and HFS → C + BF rats, (Figure 11A). We also assessed the number of iba-1 positive cells, and classified them as inactivated or activated according to previous descriptions (Rodriguez-Callejas et al., 2016). The percentage of inactivated microglia in the PFC was lower in HFS compared to the rest of the groups, but there was an increased percentage of activated microglia in HFS and HFS → C rats compared to C, HFS + BF and HFS → C + BF rats. (Figure 11B).

## 4. Discussion

In recent years, the intestine–liver–brain axis has gained great importance due to its role in the development of neurological diseases. However, there is not enough information on the development of dietary strategies that can modify this axis and reduce or prevent these diseases. The gut–brain axis is a bidirectional system between the nervous system and the gastrointestinal tract in which gut microbiota plays a key role [19]. For this reason, it is important to know the effect of specific nutrients on gut microbiota and brain function. Furthermore, the increase in obesity in the world associated with impaired cognitive function has become a serious health problem [20]. In the last decade, it has been demonstrated that the consumption of functional foods can improve cognitive function [8,11]. However, the mechanisms of action are still under investigation. Several functional foods can modify the gut microbiota, and this may impact the interaction with the liver–brain–axis. In the present work, we selected a combination of functional foods that can reshape the gut microbiota and have beneficial effects on gut barrier function, liver metabolism, and cognitive function. Dietary intake of soy protein has been associated with an increase in *A. muciniphila*, a decrease in insulin resistance, and an increase in energy expenditure [21]. Nopal, a cactus rich in polyphenols, contains soluble and insoluble fiber and vitamin C, and its intake was demonstrated to modify gut microbiota, decrease metabolic endotoxemia, and increase memory function [11]. Chia seed oil, rich in linolenic acid [9] that can be converted to the more biologically active very long-chain (n-3) PUFAs, EPA, and DHA [22]. Turmeric root contains curcumin, as a potent antioxidant which was reported to decrease oxidative stress and normalize BDNF protein [23,24] involved in learning and memory [24].

In the present study we found that chronic consumption of high fat and high sucrose diet produced the lowest diversity in gut microbiota and a significant increase in *Blautia producta* associated with visceral fat accumulation [25]. In fact, this group showed the highest % of body fat mass and the lowest % of body lean mass and oxygen consumption, generating metabolic inflexibility. Cellular rates of CO_2_ production relative to oxygen consumption, known as the respiratory exchange ratio (RER), fluctuate between 0.7 and 1.0 and provide an approximation of mitochondrial fuel used under typical condition, as seen in the control group, and is called metabolic flexibility. A high RER (1.0) is indicative of glucose oxidation, whereas a low RER reflects predominate fat oxidation (0.7). However, in the group fed an HFS diet, the transition to the fed state was accompanied by only a marginal change in RER, indicating the incapacity of the mitochondria to change substrate. This phenomenon of blunted fuel switching is known as metabolic inflexibility, and refers to the inability to respond or adapt to conditional changes in metabolic demand [26]. Interestingly, the groups HFS + BF and HFS → C + BF significantly decreased this metabolic inflexibility. Besides that, the HFS group showed the highest serum LPS concentration (468-fold increase with respect to the control group), which was associated with a lower abundance of occludin in the intestine, decreasing the stabilization of the intestinal barrier function, and generating a leaky gut due to high permeability, as has been reported in a model of a high fat diet [27]. This, in turn, allowed the access of LPS to the circulation and thus to the activation of toll-like receptor-4 (TLR4) in the colon and liver, causing low grade inflammation and the release of pro-inflammatory cytokines, particularly TNFα via the phosphorylation of NF-κB. A high abundance of TNFα induced insulin resistance [28] leading to glucose intolerance and cognitive dysfunction [29], as we observed in the HFS group. On the other hand, in the HFS group, high concentrations of insulin stimulated the abundance of the transcription factor SREBP-1 involved in lipogenesis, increasing its target enzyme fatty acid synthase (FAS), leading to an increase in serum triglycerides.

**Figure 12:**
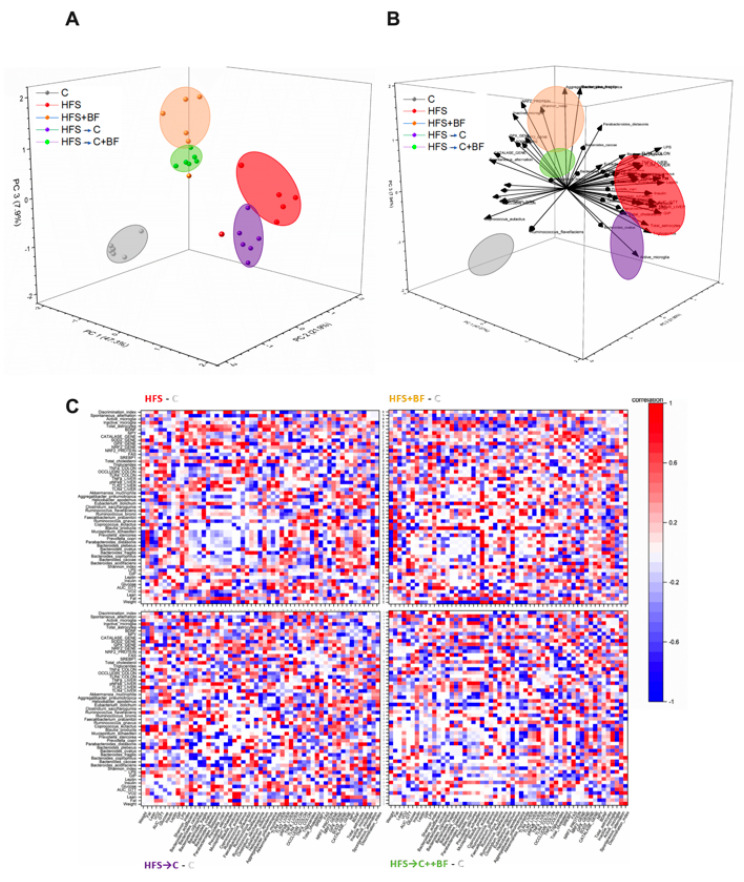
Association between biochemical parameters in liver and brain with the gut microbiota. (**A**) PCA_scores: Principal components 1, 2, and 3. Each dot corresponds to each subject, ellipse is the best fitting for each group (which includes 80% of the data). (**B**) Loading plot. Vector of each data variable on the first three principal components. Each ellipse corresponds to each group studied, (**C**) Correlation_difference. Normalized differences between correlation matrix of each group minus the C group correlation matrix of all the variables. White corresponds to the same correlation between variables, red is maximum change, and blue indicates negative difference.

This imbalance in the hepatic lipid metabolism was accompanied with low abundance of NPY in the dentate gyrus, high concentration of leptin, and a decrease in memory performance in HFS rats, associated with lower BDNF levels and reduced number of mushroom and thin spines in the PFC. Interestingly, consumption of bioactive foods was highly effective in decreasing these alterations, even in the presence of an HFS diet, an effect that was not observed when rats fed HFS returned to consume the control diet. The consumption of bioactive foods significantly increased alpha diversity of the gut microbiota by increasing a group of beneficial bacteria, particularly *P. copri, A. muciniphila, F. prausnitzii* and *B. acidifaciens*. Increased gut microbiota diversity along with the presence of these bacterial species has been associated with the intake of plant-rich diets [30,31], low grade inflammation, improved insulin sensitivity and glucose homeostasis [32], anti-inflammatory effect inhibiting NF-κB, butyrate production [33], weight control, and decreased body fat mass [34]. However, if we consider the 55 variables studied, a significant association (Figure 12C) was observed between the consumption of bioactive foods with the increase in *Aggregatibacter pneumotropica*, *Bacteroides fragilis* and *Ruminococcus brommi* with the abundance of the transcription factor NF-κΒ and the antioxidant enzymes. Moreover, the consumption of bioactive foods significantly increased the presence of eicosapentanoic acid (EPA) in serum of the groups fed bioactive foods. One of the components of the bioactive foods was chia seed oil, which is rich in alpha-linolenic acid. This polyunsaturated fatty acid (PUFA) is the precursor of the very-long chain fatty acids EPA and DHA. These omega-3 PUFAs exhibit neuroprotective properties and represent a potential treatment for a variety of neurodegenerative and neurological disorders [35]. Interestingly, in our study, only the group fed HFS + BF showed the presence of small amounts DHA (2.7 µg/µL). Several studies have demonstrated that higher plasma EPA was associated with lower gray matter atrophy of the hippocampal and parahippocampal area and amygdala in subjects aged ≥ 65 years, and with slower cognitive decline. This association did not exist for DHA [36].

The groups HFS + BF and HFS → C + BF showed higher BDNF and less neuroinflammation than the group fed HFS diet. BDNF is a neurotrophic factor that has been associated with higher energy expenditure [37]. Here, we show that lower energy expenditure, the lower BDNF and the higher leptin concentration in the HFS group were reversed when the animals were fed the HFS → C diet or with the addition of bioactive foods to the diet, indicating that the consumption of bioactive foods in an adequate diet is important to maintain better brain function.

The groups HFS + BF and HFS + C + BF also presented a significant abundance of the transcription factor NFR-2 involved in the regulation of the antioxidant enzymes SOD2, catalase, and GPx in the liver. It has been demonstrated that consumption of antioxidants prevents cognitive dysfunction [38] and reduces neuroinflammation [31] as we observed in this study. Our findings support a novel dietary therapeutic strategy to modify the gut–liver–brain axis to decrease cognitive dysfunction and metabolic abnormalities observed in liver and gut microbiota.

## 5. Conclusions

Consumption of bioactive foods resulted in an increase in the relative abundance of specific bacteria in the gut microbiota, as well as in the abundance of intestinal occludin and hepatic antioxidant enzymes. In addition, systemic inflammation and neuroinflammation decreased, and BDNF in the CPF and NPY in the hippocampus increased; these factors being the most influential in the metabolic response to bioactive food consumption according to principal component analysis (Figure 12B). As a consequence, this leads to beneficial effects on brain function, increased lean body mass, and increased oxygen consumption. With this evidence, new dietary strategies should be developed to improve the gut–liver–brain axis and cognitive function in humans.

## Data Availability

Data are available upon request.

## Appendix A

**Table A1:** Composition of experimental diets (g/100 diet).

Ingredients (%)	C	HF	HF-C	HF + BF
Cornstarch	39.77	23.90	39.77	38.7
Casein (<85% protein)	20.00	24.00	20.00	24.00
Dextrinized cornstarch	13.20	10.27	13.20	10.27
Sucrose	10.00	7.78	10.00	7.78
Soybean oil	10.00	7.00	10.00	7.00
Fiber celluose	5.00	5.00	5.00	-
Mineral mix (AIN-93MX)	3.50	3.50	3.50	3.50
Vitamin mix (AIN-93-VX)	1.00	1.00	1.00	1.00
L-Cystine	0.30	0.30	0.30	0.30
Choline bitartrate (41.1% coline)	0.25	0.25	0.25	0.25
Tert-butylhydroquinone	0.0014	0.0014	0.0014	0.0014
Lard	-	17	-	17
Chia oil	-	-	-	3
Nopal	-	-	-	5
Soy protein	-	-	-	20
Turmeric	-	-	-	1
